# A Jungle Hospital

**Published:** 1917-08-25

**Authors:** 


### A JUNGLE HOSPITAL.

### The Travelling Dispensary Officer's Work.—I.

The first duty of travelling dispensaries is to travel, and when they were inaugurated in the United Provinces of India in 1911 it was arranged that, as far as possible, they should never do that through those parts of a district already ministered to by the offshoot dispensaries from headquarters. On the contrary, they were to be free to go and give their services in those parts of a district that were far from any other form of medical aid. The *personnel* consists of the I.M.S. officer in charge and two or more native sub-assistant surgeons appointed to work under his direction. The equipment comprises a sufficiency of drugs, the instruments necessary for those operations which it is possible to perform, and a sterilising apparatus. In addition, each officer is supplied with an office tent, and, for his own use, two large tents and generally a smaller one. The two objects that the medical officer in charge of a travelling dispensary has to keep in view are, firstly, the treatment of disease in general, and, secondly, the treatment of plague in particular and the organisation and study of preventive measures.

### The "Plague Sahib."

For years plague has ravaged India in the spring and early summer, so it is natural enough that an English doctor, going among these plague-stricken people telling them of the advantages of inoculation and cleanliness in fighting the disease, should himself be known as the "Plague Sahib." The greater part of the hot-weather work of the travelling dispensary officer is organisation and inspection. The sub-assistant surgeons have to be visited constantly and their work examined and controlled, and the medical officer himself remains in camp for part of each month devising preventive schemes for the following season, and watching carefully for any signs of a fresh outbreak of plague.

### Life in the Jungle.

With the oncoming of the cold weather the most interesting work begins. As soon as the September rains are over and the cooler days of October set in, the Plague Sahib and his family leave their pleasant station bungalow and begin a nomad life in the jungle. This lasts till the dusty winds and hot nights of March drive them, always unwillingly, to seek again the shelter of a house and the ameliorations of punkahs and ice. Before starting out for the winter's camping information is sent to native men of influence giving an approximate date when the travelling dispensary camp will be in their part of the district. They, in their turn, tell the headmen of villages and the native police and Government servants, and so the news of its arrival reaches the people.

At the first stopping-place we never failed to find a row of "bimaris," or sick persons, sitting on the borders of the encampment, waiting with Eastern patience for the Doctor Sahib to come and speak to them. One could feel only compassion for them—they were so poor, and many of them so ill, and all of them so grateful for all that was done. As the bullock-carts conveying the tents and baggage could only travel from ten to twelve miles a day, and the destination of our first long halt was generally three or four marches from our headquarters, we never stopped more than a night, or perhaps two, until we had reached the spot, generally a shady grove of mango-trees, chosen for the first jungle hospital.

In the freshness of the early morning, after a substantial "chota hazri," we would leave our last camp, and, making a détour, motor slowly along the wonderful Indian roads that are like avenues, where trees meet overhead, passing through villages large and small, whose chilled inhabitants were slowly awaking to the work of a new day as the misty gold of sunrise flooded the level fields. On our arrival we would find breakfast laid under the trees, and the many activities of a first day in camp in full swing. The white canvas of the tents sent on by the bullock-cart the previous night gleams through the shady grove; the "bhisti" with his red kamarband is filling his leather "mussack" at the well; and in the distance the cook can be seen bending over his little fire of sticks, and the ayah in her scarlet coat and white chuddur comes slowly from the little row of servants' tents to lift out and take charge of the sleepy children.

### The Day's Work.

After breakfast the office claims the doctor's first moments, but soon he is among the little crowd of sick, some of them indifferently protected from the chilly winter air by brown blankets thrown around them. Others, less fortunate, have only a covering of dirty cotton. After a few sympathetic and cheery words to them, the real work of the day begins. The patients are examined in turn, and, wherever possible, relief is given. Quinine and other drugs are provided for them, antiseptics are applied, and sores dressed. Those who require treatment or care beyond the powers of the travelling dispensary are sent, when willing, to the Civil Hospital at headquarters.

The operation for cataract is the one most frequently performed by the officer in charge of a travelling dispensary. An astonishing number of the Indian people suffer from it, and it is pathetic to see their joy when they are told that they will be given back a measure of their sight, and their gratitude when the operation has been successfully performed. As each cataract patient has to spend about ten days in camp, accommodation has to be provided. This is done by the erection in a suitable position of a number of "chapars" or huts, made of a strong grass. Each caste represented among the patients is given a chapar, and generally four or five patients share the same one. Room is also found for relatives and friends who may have come with the very old or infirm.

No operations are performed on the first day in a new camp, but early on the following morning the doctor is at work. An operating-table is arranged under the shade of a tree, or in the lee of some building, occasionally on the verandah of one of the old disused bungalows which are found dotted over the United Provinces, and which date back to the prosperous days of opium and indigo. Here, all day long till the light fails, the doctor and his assistants work, sterilising the instruments, applying a local anæsthetic, and removing the cataract. Gradually the chapars fill, and by evening another line of newcomers is waiting on the outskirts of the camp, to be seen that night and operated on as soon as possible. The majority of the patients are elderly, and are of both sexes, old men with lined and hardened faces, whose hopeless expressions give place to those of wonder and thankfulness when they look up and see the trees and sky once more; Mahomedan women in their poor and dirty garments; Hindu widows with close-cropped hair, and here and there a younger man or woman whom the disease has attacked at an earlier age. They are poor and dirty and illiterate, and yet to these dwellers in the jungle, who can in no way repay its care and thought for them, the British Raj extends its just protection and its ministry of healing.

The operations and other medical work of the camp may occupy many days, but in the midst of them time must be given to the other side of the travelling dispensary work—namely, the combating of plague.

(*To be continued.*)

